# Targeting unfolded protein response reverts ER stress and ER Ca^2+^ homeostasis in cardiomyocytes expressing the pathogenic variant of Lamin A/C R321X

**DOI:** 10.1186/s12967-023-04170-y

**Published:** 2023-05-22

**Authors:** Giusy Pietrafesa, Roberta De Zio, Simona Ida Scorza, Maria Francesca Armentano, Martino Pepe, Cinzia Forleo, Giuseppe Procino, Andrea Gerbino, Maria Svelto, Monica Carmosino

**Affiliations:** 1 Department of Sciences, University of Basilicata, Potenza, Italy; 2 Department of Biosciences, Biotechnologies and Environment, University of Bari Aldo Moro, Bari, Italy; 3 Cardiology Unit, Department of Emergency and Organ Transplantation, University of Bari Aldo Moro, Bari, Italy

**Keywords:** Cardiomyocytes, ER stress, Lamin A/C, Cardiomyopathy, Guanabenz, Empagliflozin

## Abstract

**Background:**

We previously demonstrated that an Italian family affected by a severe dilated cardiomyopathy (DCM) with history of sudden deaths at young age, carried a mutation in the *Lmna* gene encoding for a truncated variant of the Lamin A/C protein (LMNA), R321X. When expressed in heterologous systems, such variant accumulates into the endoplasmic reticulum (ER), inducing the activation of the PERK-CHOP pathway of the unfolded protein response (UPR), ER dysfunction and increased rate of apoptosis. The aim of this work was to analyze whether targeting the UPR can be used to revert the ER dysfunction associated with LMNA R321X expression in HL-1 cardiac cells.

**Methods:**

HL-1 cardiomyocytes stably expressing LMNA R321X were used to assess the ability of 3 different drugs targeting the UPR, salubrinal, guanabenz and empagliflozin to rescue ER stress and dysfunction. In these cells, the state of activation of both the UPR and the pro-apoptotic pathway were analyzed monitoring the expression levels of phospho-PERK, phospho-eIF2α, ATF4, CHOP and PARP-CL. In addition, we measured ER-dependent intracellular Ca^2+^ dynamics as indicator of proper ER functionality.

**Results:**

We found that salubrinal and guanabenz increased the expression levels of phospho-eIF2α and downregulated the apoptosis markers CHOP and PARP-CL in LMNA R321X-cardiomyocytes, maintaining the so-called adaptive UPR. These drugs also restored ER ability to handle Ca^2+^ in these cardiomyocytes. Interestingly, we found that empagliflozin downregulated the apoptosis markers CHOP and PARP-CL shutting down the UPR itself through the inhibition of PERK phosphorylation in LMNA R321X-cardiomyocytes. Furthermore, upon empagliflozin treatment, ER homeostasis, in terms of ER ability to store and release intracellular Ca^2+^ was also restored in these cardiomyocytes.

**Conclusions:**

We provided evidence that the different drugs, although interfering with different steps of the UPR, were able to counteract pro-apoptotic processes and to preserve the ER homeostasis in R321X LMNA-cardiomyocytes. Of note, two of the tested drugs, guanabenz and empagliflozin, are already used in the clinical practice, thus providing preclinical evidence for ready-to-use therapies in patients affected by the LMNA R321X associated cardiomyocytes.

## Introduction

Lamin A/C (LMNA) are structural proteins of the nuclear envelope (NE) with a crucial role in cardiomyocytes function [1, 2]. As a matter of fact, a cardiac involvement associated with mutations in the *Lmna* gene was one of the first phenotypes to be reported in humans [3].

Mutations in the *Lmna* gene segregate with several cardiac phenotypes, including cardiac conduction disturbance, atrial or ventricular tachyarrhythmias, and dilated cardiomyopathy (DCM), resulting in heart failure or sudden cardiac death [4].

Cardiac laminopathies represent indeed a medical emergency being sudden cardiac death a burden impacting not only the way of life of patients and relatives but also health policies and interventions. To date, no specific therapeutic strategies are available to modify the disease progression. In fact, the clinical management of *Lmna*‐related cardiomyopathy is no different from that for other forms of dilated cardiomyopathy and the implantable cardioverter-defibrillator (ICD) represents so far the unique strategy to prevent sudden death. Different disease models have been used in the field of cardiac laminopathies and several hypotheses have been postulated to underlying the electrical and structural abnormalities in the heart of laminopathy patients [5–7]. Although the relevant advances in the knowledge of the mechanisms altering cardiac functions and structures in cardiac laminopathies, few therapeutic approaches have been proposed and developed.

The study of the Lmna^H222P/H222P^ knock-in mouse, a murine model that recapitulates the dilated cardiomyopathy in humans, demonstrated upregulation of genes implicated in the MAPK signaling pathways [8]. Accordingly, MEK1/2 inhibitors have been able to slow-down left ventricular dilatation progression, to improve cardiac contractility and functions and to increase survival of treated Lmna^H222P/H222P^ mice [9–11]. These findings led to the first clinical trial, still on going, on the p38α inhibitor in patients with LMNA-associated DCM (clinicaltrials.gov #NCT02057341).

We also found that the severity of the clinical manifestations in cardiac laminopathy patients harboring different pathogenic *Lmna* variants, correlates with the degree of inflammation in terms of numbers of pro-inflammatory cytokines upregulated, thus suggesting an immediate therapeutic approaches for each subset of patients [12].

Moreover, we recently showed that a well-known FDA-approved alkaloid, colchicine, recovered the altered tubulin polymerization state as well as the dysfunctional electrical properties in cardiomyocytes expressing a pathogenic LMNA variant [13].

The study of the pathogenic mechanisms underlying cardiac laminopathies is, indeed, an unique opportunity to identify pharmacological targets and hopefully ready-to-use therapies for these inherited cardiomyopathies.

Different cohort studies revealed that, compared with missense mutation carriers, nonsense mutation carriers had a significantly worse prognosis because of earlier onset of cardiac conduction disturbance (CCD), atrial arrhythmias, and left ventricular systolic dysfunction [14, 15]. This finding suggests that the pathogenetic mechanism of the cardiomyopathy in the nonsense mutation group might be related to either the dominant negative effect or cytotoxic effect of the resulting truncated LMNA protein rather than exclusively haploinsufficiency of LMNA wild type. Accordingly, we and others demonstrated that truncated pathogenic variants of LMNA were expressed into the heart of the carriers and may induce abnormal degradation of the WT LMNA [16] or interfere with cardiomyocytes homeostasis [13, 17, 18].

Specifically, we previously characterized nonsense *Lmna* mutation that introduces a premature termination codon within the 6th of 12 *Lmna* exons corresponding to a truncated variant in the central a-helical coiled-coil rod domain (coil 2B) of the LMNA protein, R321X. We identify this mutation in several members of an Italian family with a frequent history of sudden death and a severe DCM, confirming that this mutation is associated with a very severe cardiac phenotype and poor prognosis [17].

We have been able to detect the expression of LMNA R321X in the transplanted heart of a patient carrying this nonsense mutation. When we tried to get more insights into the disease-causing mechanisms by heterologous expression of LMNA R321X, we found that R321X was not targeted to the nuclear envelope, as expected by the absence of the nuclear localization sequence in the truncated version of LMNA, rather it accumulates in the ER. Functional studies showed that the presence of R321X into the ER caused the onset of the ER stress and the unfolded protein response (UPR). The latter triggers ER Ca^2+^ handling abnormalities and thus increased susceptibility to apoptosis [17].

The UPR is a complex signal transduction pathway that is initiated by the activation of at least three UPR stress sensors: inositol-requiring protein 1 (IRE1), protein kinase RNA-like ER kinase (PERK) and activating transcription factor 6 (ATF6). UPR signaling has distinct kinetics, intensities and downstream consequences depending on the nature and intensity of the stimuli and the cell type involved [19]. In this work we provided evidence that in cardiomyocytes the expression of LMNA R321X variant induced specifically the activation of the PERK arm of the UPR and that targeting this pathway we have been able to rescue the functional phenotype of these cardiomyocytes in terms of ER function and cell survival.

## Methods

### Cell culture

HL-1 cardiomyocytes were cultured on gelatin/fibronectin coated flasks (5 μg/mL fibronectin, F1141, Sigma-Aldrich, in 0.02% Gelatin from bovine skin, G9391, Sigma-Aldrich) using Claycomb Medium (51800C, Sigma-Aldrich) supplemented with 10% fetal bovine serum (F2442, Sigma-Aldrich), Penicillin/Streptomycin 100 U/mL: 100 µg/mL (P4333, Sigma-Aldrich), 2 mM l-Glutamine (G7513, Sigma-Aldrich) and 0.1 mM Norepinephrine [( ±)-Arterenol] (A0937, Sigma-Aldrich) in a humidified 5% CO_2_, 95% O_2_ incubator at 37 °C.

Cell concentration and viability were assessed using Trypan Blue Stain, 0.4% with LUNA-II™ Automated Cell Counter (Logos Biosystems).

### Generation of stable HL-1 clones expressing either WT or R321X LMNA

Lentiviral construct coding for LMNA R321X mCherry-tagged protein was produced by Stratagene's Quik Change II XL site-directed mutagenesis kit (Agilent Technologies), using the the lentiviral plasmid encoding for mCherry-tagged Lamin WT mCherry-tagged (pLV[Exp]-Neo-CMV > mCherry(ns):hLMNA[NM_170707.4], Vector Builder). Mutagenic primers were designed using Quik Change Primer Design Program as previously shown [17]. The mutation was verified by sequencing.

Viral particles were produced in HEK-293T cells by simultaneously co-transfection (Invitrogen™ Lipofectamine™ 2000 Transfection Reagent) of the plasmid carrying the gene for protein of interest (mCherry-tagged-Lamin WT or mCherry-tagged-LMNA R321X), the envelope plasmid encoding VSV-G and the packaging plasmid encoding Gag/Pol and Rev, as previously reported [13].

For lentiviral transduction, HL-1 cells were plated in a 6-well plate at 30–50% of confluency, and after adhesion, they were incubated with 500 µL of virus-containing medium at 37 °C in a humidified 5% CO_2_ incubator for 18 h. After viral particles removal, cells were then incubated with 400 μg/mL Geneticin (G418, Gibco, Life Technologies) for 1 week to select stable clones. Pure clones were selected by fluorescence microscopy.

### Immunofluorescence confocal analysis

Stable transduced HL-1 cells were grown on coverslips to confluency, then were fixed in PFA with 0.1% Triton X-100 for 20 min at RT. After washes with PBS, cells were blocked in 1% bovine serum albumin (BSA) in PBS for 30 min at room temperature (RT) and incubated with anti-Calnexin (dil. 1:500; Santa Cruz Biotechnology). After three washes in PBS cells were incubated with 488 Alexafluor-conjugated secondary antibodies (Thermo Fisher Scientific) for 1 h at RT for calnexin identification. The signal corresponding to LMNA proteins was detected using their fluorescent m-Cherry tag. After removal of the secondary antibody, coverslips were mounted in PBS/glycerol (1:1) containing 1% *n*-propylgallate, pH 8.0.

Confocal images were acquired with a confocal laser-scanning fluorescence microscope (Leica TSC-SP2).

### MTT assay

The in vitro cytotoxicity for salubrinal (SML0951, Sigma-Aldrich), guanabenz (G110, Sigma-Aldrich) and empagliflozin (S8022, Selleckchem) was verified by 3-(4,5-dimethylthizol-2-yl)-2,5-diphenyl tetrazolium bromide (MTT) colorimetric assay [20] (Sigma-Aldrich).

HL-1 cells were seeded in 96-well plates at the density of 1.0 × 10^4^ cells/well. After complete adhesion, cells were treated for 48 h in complete grow medium with the appropriate compounds at three different concentrations (salubrinal: 100, 200, 300 µM; guanabenz: 10, 20, 30 µM; empagliflozin: 5, 10 e 50 µM). Cells were maintained in a humidified incubator (at 37 °C, 5% CO_2_) throughout the treatment, refreshing the medium containing the proper amount of drugs after 24 h of incubation. Cells in complete medium without treatments were used as healthy control cells, whereas 0.1% Triton x-100 for 4 h was used as positive control of cellular death.

At the end of the 48-h treatment, 5 mg/mL MTT were added to the cells and incubated for 3 h. Next, the cells were washed two times with PBS to remove detached dead cells and the formazan crystals were solubilized adding 100 µL of DMSO to each well.

Absorption was detected at 595 nm with a microplate reader (Bio-Rad) and corrected for background absorption at 620 nm.

The cytotoxicity of the drugs used in our experiments was determined by statistically comparing the absorbance measured in treated and untreated control cells.

### Cytosolic Ca^2+^ measurements

For intracellular Ca^2+^ measurements, HL-1 cardiomyocytes, stably expressing WT or LMNA R321X protein were plated on gelatin/fibronectin coated 15‐mm coverslip 96 h before the experiment. After complete cell adhesion, cells were left in complete Claycomb media or incubated with 200 µM salubrinal, 20 µM guanabenz or 10 µM empagliflozin added to the complete Claycomb media for a 48 h in a humidified incubator (5% CO_2_, 95% O_2_) at 37 °C.

Ca^2+^ measurements were performed using 6 μM Fura-2, AM (F1221, Invitrogen). Cells were dye-loaded for 30 min at 37 °C in medium before the experiment. After the incubation, cells were washed with extracellular ringer (138 mM NaCl, 4 mM KCl, 1 mM MgCl_2_, 10 mM Hepes, 10 mM Glucose, 1.8 mM CaCl_2_, pH 7.4) and left in the incubator to allow de-esterification of the dye. Coverslips with dye-loaded cells were mounted in a perfusion chamber (RC quick release non-magnetic chamber, Warner Instruments), housed in a Quick Exchange Platform (QE-1, Warner Instruments) and analyzed using an inverted microscope (Nikon Eclipse TE2000-S microscope) equipped for single cell fluorescence measurements and imaging analysis. The sample was illuminated through a 40 × oil immersion objective (NA = 1.30). The Fura-2AM loaded sample was excited alternately at 340 and 380 nm every 5 s. Emitted fluorescence was passed through a dichroic mirror, filtered at 510 nm (Omega Optical) and captured by a cooled CCD camera (CoolSNAP HQ, Photometrics). Cells were stimulated with 40 µM cyclopiazonic acid (CPA, C1530, Sigma-Aldrich) in Ca^2+^-free extracellular solution containing 50 µM EGTA (E4378, Sigma-Aldrich) to induce ER Ca^2+^ depletion. After 20 min of stimulation with CPA, re-addition of extracellular Ca^2+^ (5 mM Ca^2+^) in the continuous presence of CPA induced capacitative calcium entry (CCE). Fluorescence measurements were performed using Metafluor software (Molecular Devices, MDS Analytical Technologies). The fluorescence ratio was recorded and normalized to the basal fluorescence ratio observed in the absence of stimulus (R/R0). Experiments were performed at room temperature.

For determination of intracellular Ca^2+^ concentration, Fura-2AM traces were corrected for the background fluorescence and fluorescence ratio (R) was calibrated according the following equation: [Ca^2+^]_i_ = Kd × Q(R − Rmin)/(Rmax − R), where Kd (224 nM) indicated the dissociation constant of Fura-2AM for Ca^2+^_i_ and Q indicated the ratio of the fluorescence intensities at the minimum and the maximum Ca^2+^ concentration at 380 nm. Each sample was calibrated by the addition of 5 µM ionomycin in presence of 0.5 mM EGTA (Rmin) followed by 5 µM ionomycin in 10 mM CaCl_2_ (Rmax).

### Evaluation of NFAT-GFP translocation

The evaluation of NFAT translocation was performed as previously described [21]. Briefly HL-1 cells were transfected with NFATc4-GFP (kindly provided by Chi-Wing Chow (Department of Molecular Pharmacology, Albert Einstein College of Medicine, Bronx, NY, USA) with Lipofectamine 2000 according to the manufacturer’s instructions. After 24 h, cells were either left untreated (CTR) or stimulated for 48 h as follows: 200 µM salubrinal, 10 µM guanabenz, 10 µM empagliflozin, in a humidified incubator at 37 °C, 5% CO_2_, and 95% O_2_.

Cells were fixed in PBS containing 4% paraformaldehyde and stained with DAPI for nuclear counter staining. Ten different fields for each treatment from three independent experiments were acquired blindly using the Leica DM6000B fluorescence microscope (Leica Instruments). Cells expressing NFAT-GFP were counted, and Image J was used to keep track of the nuclei vs cytosol-positive staining of the probe using DAPI colocalization to confirm NFAT nuclear translocation.

### Western blot analysis

For protein extraction HL-1 LMNA clones were plated in a 12 wells cell culture support coated with gelatin-fibronectin until 80% of confluency. Cells were then left in complete Claycomb medium or treated adding to complete Claycomb medium either 200 µM salubrinal, 10 µM guanabenz or 10 µM empagliflozin for 48 h in a humidified incubator (5% CO_2_, 95% O_2_) at 37 °C.

After treatments cells were placed on ice, washed in PBS and lysed in RIPA buffer added with protease and phosphatase inhibitors (NaCl 150 mM, Tris/HCl 10 mM, 1% Triton X‐100, SDS 0.1%, deoxycholate‐Na 1%, EDTA 5 mM; NaF 10 mM, orthovanadate 100 mM, pyrophosphate 15 mM, pH 7.2). Cell suspensions were sonicated 3 times for 15 s at 60 Ampli with a Vibra‐cell^®^ (Sonics & Materials Inc) and membrane debris were pelleted at 13,000 rpm for 30 min at 4 °C. Samples were quantified by Bradford protein assay and set up for the western blot analysis in 4 × Laemmli Sample Buffer (Bio-Rad) plus 50 mM DTT.

Protein samples were denatured at 60 °C for 10 min, resolved by electrophoresis on polyacrylamide SDS gel (7.5% or 12% Mini-PROTEAN TGX Stain-Free Precast Gels; Bio‐Rad) and blotted on 0.2 µm PVDF membrane (Trans-Blot Turbo RTA Mini 0.45 µm LF PVDF Transfer Kit, for 40 blots #1704274, Bio-Rad) through the Trans-Blot Turbo Transfer System (Bio‐Rad). Immobilized proteins were blocked at room temperature for 1 h in TBST-5% milk or in TBST‐BSA 3% (TBST: 50 mM Tris, 150 mM NaCl, 0.1% Tween‐20; pH 7.4) and incubated overnight at 4 °C with the following antibodies prepared in blocking buffer: anti-PARP (dil. 1:1000 in TBST-5% milk; #9542 Cell Signaling Technology), anti-Phospho-eIF2α (Ser51, dil. 1:1000 in TBST‐BSA 5%; #9721 Cell Signaling Technology), anti-CHOP (dil. 1:1000 in TBST‐BSA 5%; #2895 Cell Signaling Technology), anti-XBP-1s (dil. 1:1000 in TBST-5% milk; #647501 BioLegend), anti-phospho-PERK (Thr982, dil. 1:1000 in TBST-5% milk; #SAB5700521 Sigma Aldrich), anti-GRP78/BiP (dil. 1:1000 in TBST‐5% BSA; #ab21685 Abcam), anti-ATF4 (dil. 1:1000 in TBST-5% milk; #11815 Cell Signaling), anti-STIM1 (dil. 1:1000 in TBST-5% milk; #66189-1 Proteintech), anti-ORAI1 (dil. 1:5000 in TBST-5% milk, #66223-1 Proteintech), anti-Na^+^/K^+^-ATPase α subunit (dil. 1.1000 in TBST- 1% BSA; # C464.6 Millipore), anti-phospho-AKT (Ser473; dil. 1:4000 in TBST‐1% BSA; #4060 Cell Signaling).

Secondary antibodies were selected depending on the host species of the primary antibodies and prepared in the relative blocking buffer (Goat anti-mouse IgG‐HPR conjugate 1:5000, Bio‐Rad; Goat anti‐rabbit IgG‐peroxidase 1:5000, Sigma‐Aldrich). Chemiluminescent reactions were detected thanks to ChemiDoc™ System (Bio‐Rad) using ECL substrate (Clarity western ECL substrate #1705061 or Clarity max Western ECL Substrate #1705062, Bio-Rad). Densitometric analysis was performed by Image Lab 6.0 software. Stain‐free technology (Bio‐Rad) allowed quantifying protein loading.

### Statistical analysis

For both intracellular Ca^2+^ measurements and western blot analysis data are given as mean ± standard error of the mean (SEM). Student’s T-test and One-way Anova were used to compare different experimental conditions. Significance was accepted for p-values < 0.05. The software GraphPad Prism 8 allowed performing the statistical analysis and graphical representation of the data.

## Results

### Activation of the PERK arm of the UPR in LMNA R321X-expressing cardiomyocytes

**Fig. 1:**
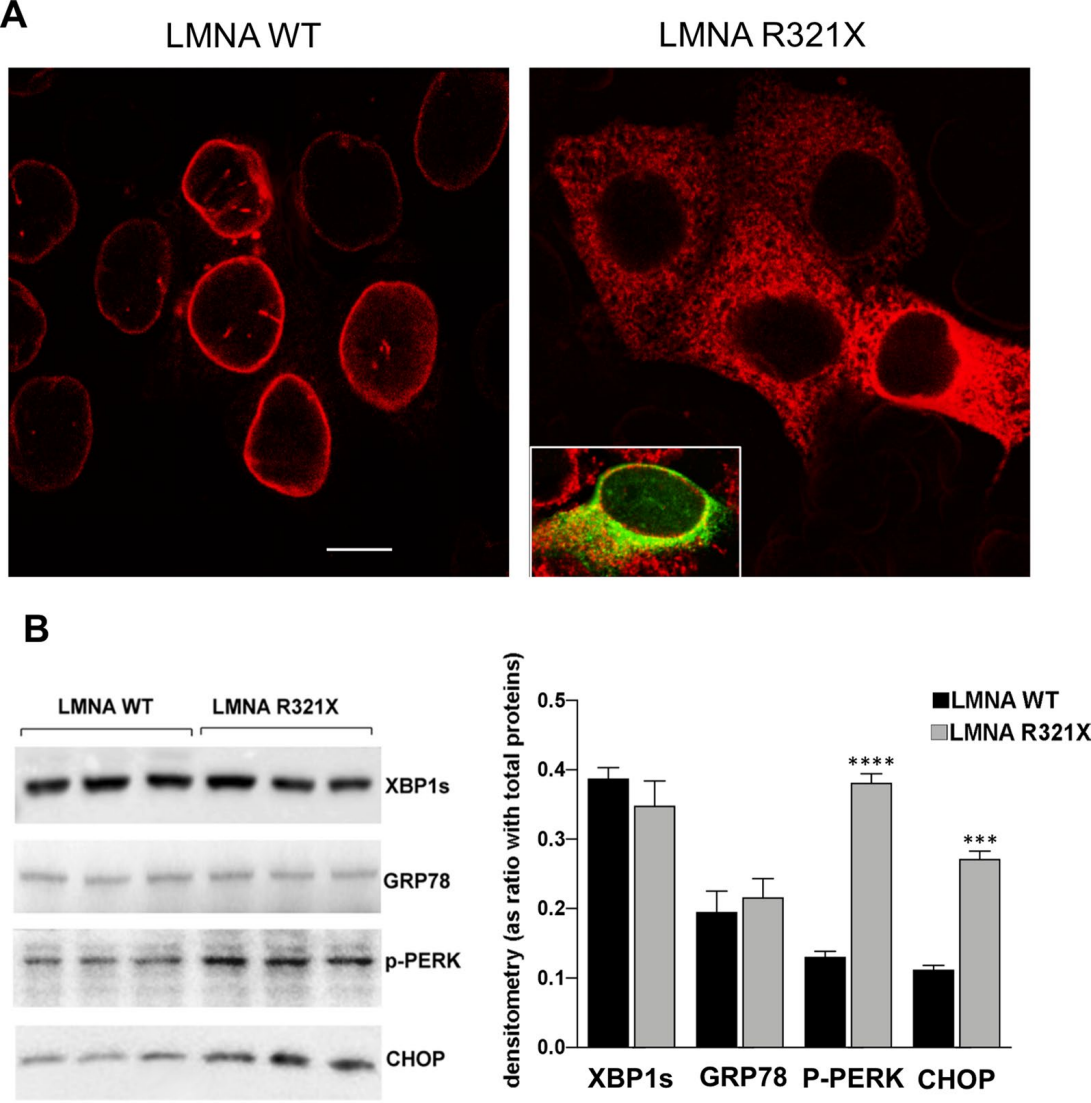
Expression of WT and R321X LMNA in HL-1 cardiomyocytes. **A** Confocal laser fluorescence analysis of LMNA in HL-1 clones where red signals correspond to m-Cherry tagged LMNA proteins and the green signal, in the inset, corresponds to the ER marker calnexin. Bar = 10 µm. **B** Expression of ER stress markers in WT and R321X LMNA-cardiomyocytes. Left panel: representative Western blotting of XBP1s, GRP78, p-PERK and CHOP in the two lines of cardiomyocytes (LMNA WT, LMNA R321X). The triplicate of each experimental condition in the representative western blotting corresponds to three different biological replicas. Right panel: densitometric analysis of the immunoreactive bands corresponding to the ER stress markers, normalized for total protein content. Data are represented as mean ± SEM. Statistical analysis was performed on three independent experiments and significance calculated by Student's *t*-test for unpaired samples. (****p = 0.0001, LMNA R321X vs LMNA WT; ***p = 0.0002, LMNA R321X vs LMNA WT)

We generated HL-1 cardiomyocytes stably expressing LMNA R321X to recapitulate the cellular phenotype of R321X-cardiolaminopathy. Control LMNA WT-cardiomyocytes were generated as well. As previously shown in HEK-293 cells, LMNA R321X accumulates into the ER of HL-1 cardiomyocytes in contrast with LMNA WT, which, as expected, decorates the nuclear rim (Fig. 1A, LMNA WT, LMNA R321X). We previously demonstrated in HEK-293 cells and in R321X carrier’s heart, that R321X accumulation into the ER triggers ER stress and the activation of the PERK-CHOP branch of the UPR [17]. During ER stress, PERK activation induces the phosphorylation and inhibition of the eukaryotic translation initiation factor 2 alpha (eIF2α), thus reducing the global protein synthesis and consequently ER workload. On the other hand, phosphorylation of eIF2α increases the cap-independent translation of certain mRNAs, such as activating transcription factor 4 (ATF4). ATF4 is a transcription factor that regulates a wide range of genes playing a crucial role in cell adaptation to stress conditions and cell survival, but, during long-term ER stress, ATF4 may also stimulate genes of CCAAT-enhancer-binding protein homologous protein (CHOP), which is responsible for initiation of the apoptotic cascade (for review see [19]). As shown in Fig. 1B, the expression of LMNA R321X in cardiomyocytes significantly increased the expression of both phospho-PERK (p-PERK) and CHOP compared to their controls, thus confirming the activation of PERK-CHOP pathway. We also checked for the expression of spliced X-box-binding protein-1 (s-Xbp1) and of (Glucose-Regulated Protein 78) GRP78 proteins whose expression levels are associated with the activation of IRE1α- and ATF-6-dependent pathways of the UPR response [22], respectively. As shown in Fig. 1B their expression was comparable in the two lines of cardiomyocytes.

### Targeting eIF2α in LMNA R321X- cardiomyocytes: effect of salubrinal and guanabenz

DNA damage-inducible 34 (GADD34) protein is involved in braking PERK-CHOP arm of UPR by functioning as a protein phosphatase 1 (PP1) subunit targeting phosphorylated eIF2α. Previous evidence demonstrated that inhibition of eIF2α dephosphorylation by GADD34 deletion or using small molecule inhibitors of the GADD34–PP1 complex, prolonged eIF2α phosphorylation, subsequently leading to adaptive UPR and promoting cell survival [23].

**Fig. 2:**
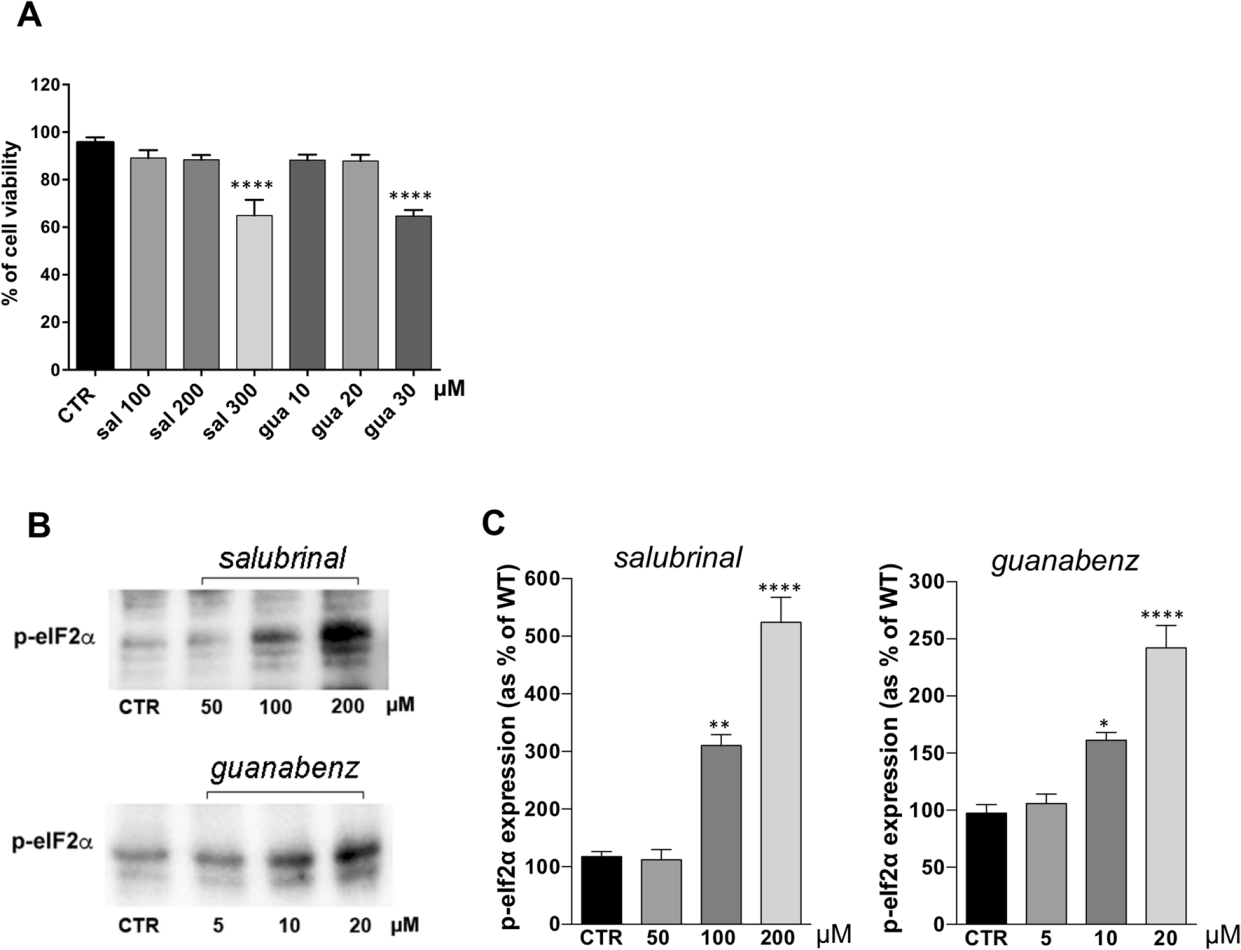
Effect of salubrinal and guanabenz on cell viability and eIF2α phosphorylation. **A** MTT assay performed on LMNA R321X-cardiomyocytes upon 48 h incubation with salubrinal and guanabenz at three different concentrations. Statistical analysis was performed on three independent experiments and significance calculated by one-way Anova. Significancy of untreated cardiomyocytes (CTR) vs treated cardiomyocytes is reported (****p = 0.0001). **B** Representative Western blotting of phospho-eIF2α (p-eIF2α) performed on lysates from LMNA R321X-cardiomyocytes treated with either salubrinal or guanabenz at three different concentrations for 48 h. The triplicate of each experimental condition in the representative western blotting corresponds to three different biological replicas. **C** Densitometric analysis of the immunoreactive bands corresponding to p-eIF2α in the experimental conditions shown in A, normalized for total protein content and reported as % of the control condition. Data are represented as mean ± SEM. Statistical analysis was performed on three independent experiments and significance calculated by one-way ANOVA and Tukey's multiple comparisons test. Significancy of untreated cardiomyocytes (CTR) vs treated cardiomyocytes is reported (*p = 0.01, **p = 0.003, ****p = 0.0001)

**Fig. 3:**
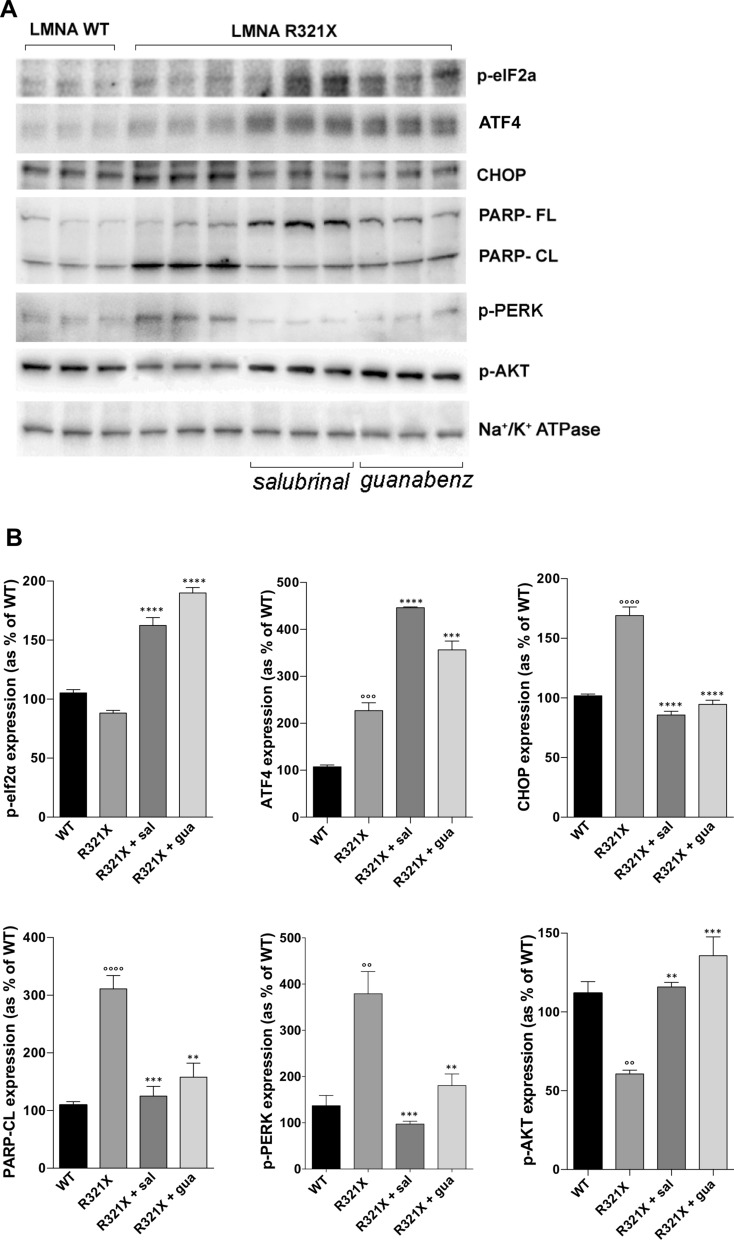
Effect of salubrinal and guanabenz on the expression of markers of PERK and AKT pathways in LMNA R321X-cardiomyocytes. **A** Representative Western blotting of phospho-eIF2α (p-eIF2α), ATF4, CHOP, full length PARP (PARP-FL) and cleaved PARP (PARP-CL), p-PERK and p-AKT performed on lysates from LMNA R321X-cardiomyocytes treated with either 200 µM salubrinal or 20 µM guanabenz for 48 h and from LMNA WT-cardiomyocytes as control. The expression of Na^+^/K^+^-ATPase α subunit was also checked in all samples as control. The triplicate of each experimental condition in the representative western blotting corresponds to three different biological replicas. **B** Densitometric analysis of the immunoreactive bands corresponding to p-eIF2α, ATF4, CHOP, PARP-CL, p-PERK, p-AKT in the experimental conditions shown in **A**, normalized for total protein content and reported as % of the control condition (WT). Data are represented as mean ± SEM. Statistical analysis was performed on three independent experiments and significance calculated by one-way ANOVA and Tukey's multiple comparisons test (°°°°p = 0.0001, °°°p = 0.0003, °°p = 0.003 for WT vs R321X; **p = 0.003, ***p = 0.0003, ****p = 0.0001 for untreated R321X vs treated R321X)

We thus tested the effect of two inhibitors of the GADD34–PP1 complex, salubrinal and guanabenz, in inducing sustained eIF2α phosphorylation without affecting cell viability in LMNA R321X-cardiomyocytes. MTT test clearly demonstrated that cell viability was not significantly affected for concentrations below 300 µM salubrinal and 30 µM guanabenz (Fig. 2A). The concentrations tested in the MTT assay have been chosen in the range of those reported in bibliography to be effective in sustaining adaptive UPR. Both compounds after 48 h of incubation at non-toxic concentrations were able to increase the phosphorylation levels of eIF2α in LMNA R321X-cardiomyocytes in a dose-dependent fashion (Fig. 2B, C, p-eIF2α). For the following experiments we used 200 µM salubrinal and 20 µM guanabenz for 48 h, since MTT test clearly demonstrated that cell viability was not significantly affected at these concentrations although the levels of p-eIF2α were significantly increased. We studied the possible beneficial effect of the sustained eIF2α phosphorylation upon salubrinal and guanabenz treatment analyzing the expression levels of the major players in the PERK branch of the UPR. As shown in Fig. 3A, B, 48 h treatment with either salubrinal or guanabenz, significantly reduced the expression of both pro-apoptotic factor CHOP, and the caspase-dependent apoptosis marker, cleaved Poly (ADP-ribose) polymerase 1 (PARP-CL), in LMNA R321X-cardiomyocytes almost at levels of LMNA WT-cardiomyocytes (Fig. 3A, CHOP, PARP-CL; Fig. 3B CHOP expression, PARP-CL expression). The efficacy of both drugs was proved by the increased levels of p-eIF2α in treated LMNA R321X-cardiomyocytes (Fig. 3A, p-eIF2α; Fig. 3B, p-eIF2α expression). As expected, the expression of p-eIF2α downstream effector, ATF4, increased together with the sustained p-eIF2α phosphorylation under salubrinal or guanabenz treatments (Fig. 3A, ATF4; Fig. 3B, ATF4 expression). Interestingly, the phosphorylation level of the ER stress sensor PERK decreased under drug treatments in LMNA R321X-cardiomyocytes although both drugs were targeting the downstream effector of PERK, p-eIF2α (Fig. 3A, p-PERK; Fig. 3B, p-PERK expression). This finding confirms that salubrinal and guanabenz were actually alleviating ER stress. It has been demonstrated that ER stress may also suppress AKT/TSC/mTOR signaling pathway [24, 25]. Accordingly we found that phosphorylated AKT (p-AKT) was significantly downregulated in LMNA R321X-cardiomyocytes compared to their controls and that both salubrinal and guanabenz re-established the levels of p-AKT at the levels of those measured in LMNA WT-cardiomyocytes (Fig. 3A, p-AKT; Fig. 3B, p-AKT expression). The expression of the house keeping protein Na^+^/K^+^-ATPase α subunit was also analyzed to confirm that overall protein synthesis was not altered by the increased levels of p-eIF2α upon salubrinal and guanabenz treatments (Fig. 3A, Na^+^/K^+^-ATPase).

### Targeting PERK in LMNA R321X-cardiomyocytes: effect of empagliflozin

The treatment with salubrinal and guanabenz was aimed at inducing an adaptive UPR pathway, which predominantly maintains the ER function and/or ER proteostasis under ER stress conditions. However, long-term or severe ER stress could activate a maladaptive UPR pathway leading to eliminate dysfunctional cells by apoptosis or autophagy. In this scenario, shutting-down the UPR response may also results beneficial for cell survival and homeostasis. The PERK-dependent UPR pathway initiates with PERK auto-phosphorylation by its kinase domain when its cytoplasmic domain senses the accumulation of unfolded/misfolded proteins in the ER lumen. Several inhibitors of PERK catalytic domain have been developed and shown to be effective in down-regulating UPR response promoting cells survival [26]. However, they resulted to have off-target effects, which limited their use in clinical practice [27].

Interestingly, empagliflozin, a selective sodium-glucose co-transporter-2 (SGLT-2) inhibitor, a new class of oral anti-diabetic agents approved for the treatment of type 2 diabetes mellitus, has been demonstrated to provide additional non-glycemic benefits, counteracting the activation of PERK-CHOP branch of the UPR in either cardiovascular or neuronal diseases [28].

**Fig. 4:**
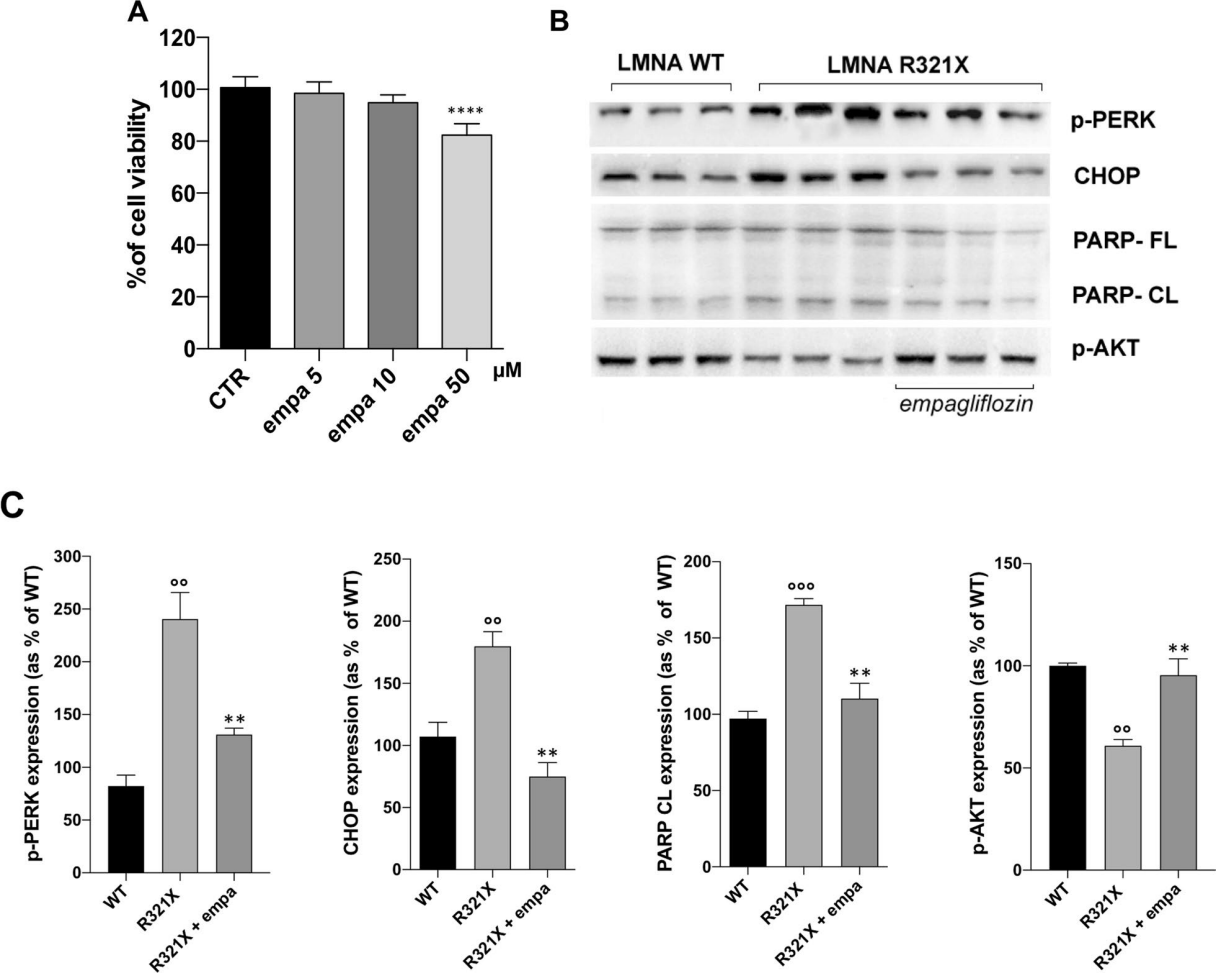
Effect of empagliflozin on the expression of markers of PERK and AKT pathway in LMNA R321X-cardiomyocytes. **A** MTT assay performed on LMNA R321X-cardiomyocytes upon 48 h incubation with empagliflozin at three different concentrations. Statistical analysis was performed on three independent experiments. Data reported as mean ± SEM and significance calculated by one-way Anova. **B** Representative Western blotting of p-PERK, CHOP, full length PARP (PARL-FL), cleaved PARP (PARP-CL) and p-AKT performed on lysates from LMNA R321X-cardiomyocytes treated with 10 µM empagliflozin for 48 h and from LMNA WT-cardiomyocytes as control. The triplicate of each experimental condition in the representative western blotting corresponds to three different biological replicas. **C** Densitometric analysis of the immunoreactive bands corresponding to p-PERK, CHOP, PARP-CL and p-AKT in the experimental conditions shown in **B**, normalized for total protein content and reported as % of the control condition (WT). Data are reported as mean ± SEM. Statistical analysis was performed on three independent experiments and significance calculated one-way ANOVA and Tukey's multiple comparisons test (°°p = 0.001 and °°°p = 0.0006 for WT vs R321X; **p = 0.007 for untreated R321X vs treated R321X)

We indeed, analyzed the possible effect of empagliflozin in LMNA R321X-cardiomyocytes in alleviating ER disfunction. First of all we performed a cell toxicity assay incubating LMNA-cardiomyocytes with empagliflozin at 3 different concentrations for 48 h. Concentrations to be tested have been chosen in the range of those reported in bibliography to be effective in counteracting UPR (Fig. 4A). In the following experiments we used 10 µM empagliflozin as the maximal dose at which cell viability was not significantly affected.

As shown in Fig. 4B, C treatment with 10 µM empagliflozin significantly decreased the expression levels of both CHOP and PARP-CL in LMNA R321X-expressing cardiomyocytes toward those observed in LMNA WT-cardiomyocytes (Fig. 4B, CHOP and PARP-CL; Fig. 4C, CHOP expression and PARP-CL expression). The downregulation of p-PERK in empagliflozin treated LMNA R321X-cardiomyocytes clearly indicated the efficacy of this drug in braking the PERK-dependent UPR (Fig. 4B p-PERK; Fig. 4C p-PERK expression). We also checked whether empagliflozin was able to rescue the phosphorylation levels of AKT in LMNA R321X-cardiomyocytes. As shown in Fig. 4 empagliflozin significantly increased p-AKT levels in treated LMNA R321X-cardiomyocytes toward those of LMNA WT-cardiomyocytes (Fig. 4B, p-AKT; Fig. 4C, p-AKT expression) confirming the ability of this drug to promote pro-survival pathways in cells and animal models [29].

### Effect of all drugs tested on ER functions

We previously demonstrated in HEK-293 cells that ER stress induced ER function impairment characterized by an increased Ca^2+^ leakage from the ER and a decreased ability to restore ER luminal levels of Ca^2+^ [17].

**Fig. 5:**
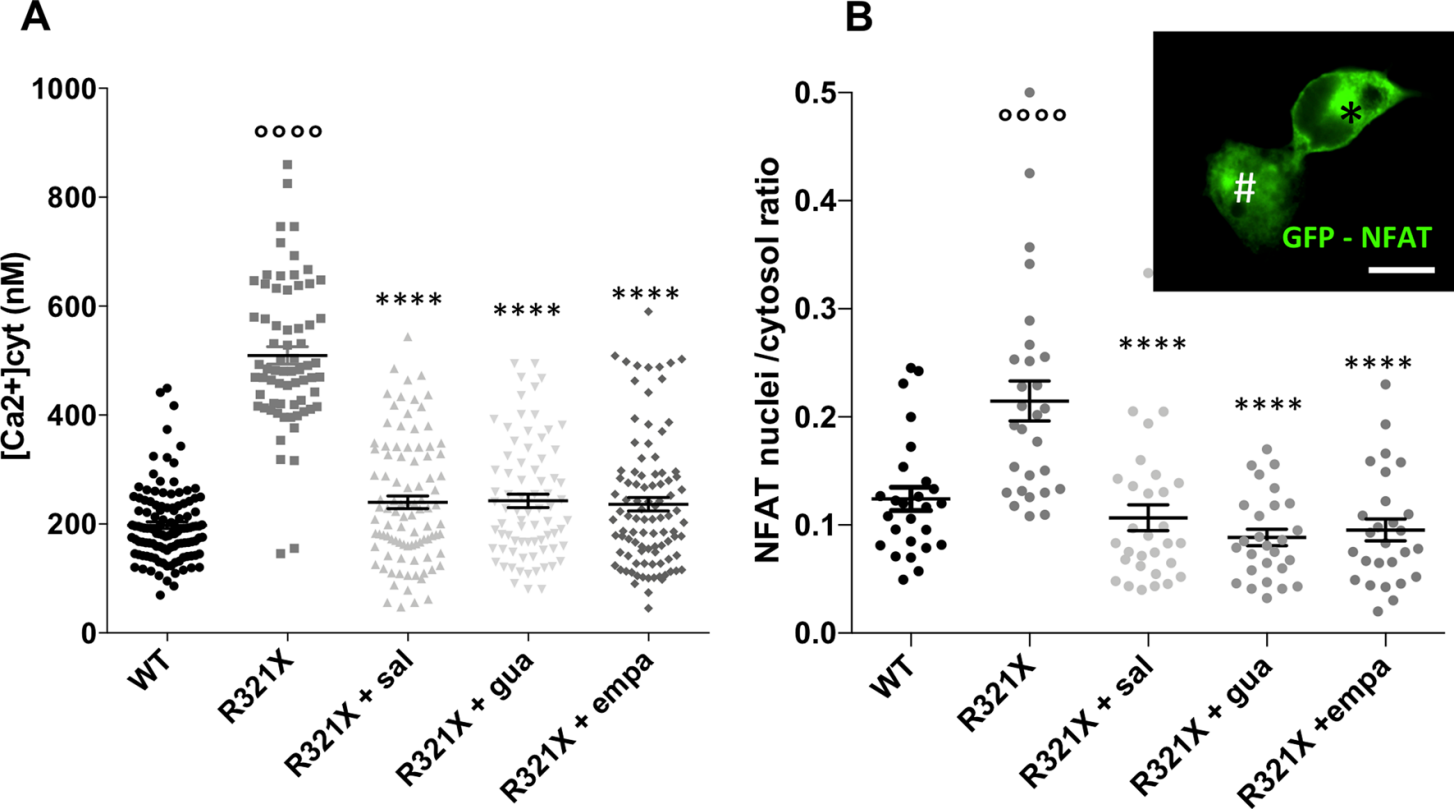
Effect of salubrinal, guanabenz and empagliflozin on [Ca^2+^]_cyt_ and NFAT nuclear translocation in LMNA R321X-cardiomyocytes. **A** Data plot summarizing analysis of the steady state [Ca^2+^]_cyt_ in LMNA WT and R321X-cardioyocytes (WT, n = 117; R321X, n = 68) and in treated LMNA R321X-cardiomyocytes (R321X + sal, n = 95; R321X + gua, n = 76; R321X + empa, n = 96). Data are reported as mean ± SEM. Statistical analysis was performed on three independent experiments and significance calculated by one-way ANOVA and Tukey's multiple comparisons test (°°°°p = 0.0001 for WT vs R321X; ****p = 0.0001 for untreated R321X vs treated R321X). **B** Data plot summarizing the ratio between the number of cells expressing NFAT-GFP in the nucleus vs cells expressing the probe in the cytosol. Inset reports representative image of HL-1 cells expressing NFAT-GFP either in the cytosol (*) or in the nucleus (#). Scale bar: 20 μm. NFAT localization was evaluated both in LMNA WT and LMNA R321X-cardiomyocytes (WT, R321X) and in treated LMNA R321X-cardiomyocytes (R321X + sal, R321X + gua, R321X + empa). Data were obtained from 3 independent experiments. Ten different fields for each treatment were blindly acquired and analyzed for every independent experiment. Cells analyzed for each experimental condition: LMNA WT *n* = 2763 cells, LMNA R321X *n* = 2548 cells, LMNA R321X + sal *n* = 1941 cells, LMNA R321X + gua *n* = 3259 cells, LMNA R321X + empa *n* = 2032 cells. The plot shows mean ± SEM and significant differences, were calculated by ordinary one-way ANOVA and Tukey's multiple comparisons test (°°°°p = 0.0001 for WT vs R321X; ****p = 0.0001 for untreated R321X vs treated R321X)

It is well known that leaky ER induces in turn a cytosolic Ca^2+^overload [30], which in cardiomyocytes could profoundly affect either electrical or contractile function. We indeed measured the cytosolic Ca^2+^ concentration [Ca^2+^]_cyt_ in LMNA WT and R321X-cardiomyocytes by the use of Fura2-AM fluorescent Ca^2+^ dye (see methods for details) and we found that in resting condition [Ca^2+^]_cyt_ in LMNA R321X-cardiomycytes was almost three fold higher than those measured in LMNA WT cardiomyocytes in which, in turn, was in the expected physiological range (Fig. 5A, WT: 197.6 ± 6.27 nM; R321X: 509.5 ± 16.16 nM). Interestingly, the treatment of LMNA R321X-cardiomyocytes with salubrinal, guanabenz or empagliflozin significantly lowered the [Ca^2+^]_cyt_ almost at levels found in controls, thus indicating that all of them were able to counteract ER Ca^2+^ leakage in these cardiomyocytes (Fig. 5A, R321X + sal: 240 ± 11.63 nM; R321X + gua: 242 ± 12.36 nM; R321X + empa: 236.2 ± 12.36).

It is well known that the increase in [Ca^2+^]_cyt_ induces the activation of the Ca^2+^/calmodulin-dependent serine/threonine protein phosphatase, calcineurin, which de-phosphorylates the nuclear factor of activated T-cells (NFAT), inducing its translocation into the nucleus [31]. Moreover, UPR response may per se activate calcineurin, since its phosphatase activity on ER chaperons may relieve ER stress [32].

We have, indeed, analyzed if calcineurin was activated in LMNA R321X-cardiomyocytes monitoring the nuclear translocation of a GFP-tagged version of NFAT (GFP-NFAT), when expressed in these cardiomyocytes both in resting condition and upon drugs treatments.

The subcellular localization of GFP-NFAT, expressed as the ratio between the number of cardiomyocytes expressing the probe in the nucleus (* in the inset of Fig. 5B) vs those expressing the probe in the cytosol (# in the inset of Fig. 5B), was compared in resting and treated LMNA R321X-cardiomyocytes and in LMNA WT-cardiomyocytes as control (Fig. 5B). Of note, we found a significant increase in NFAT-positive nuclei in LMNA R321X-cardiomyocytes, compared to LMNA WT-cardiomyocytes (Fig. 5B, R321X). The treatment of LMNA R321X-cardiomyocytes with salubrinal, guanabenz or empagliflozin restored the prevalent cytosolic localization of GFP-NFAT as shown by the ratio between cardiomyocytes expressing the probe in the nucleus vs those expressing the probe in the cytosol comparable to that of LMNA WT-cardiomyocytes (Fig. 5B, R321X + sal, R321X + gua, R321X + empa).

**Fig. 6:**
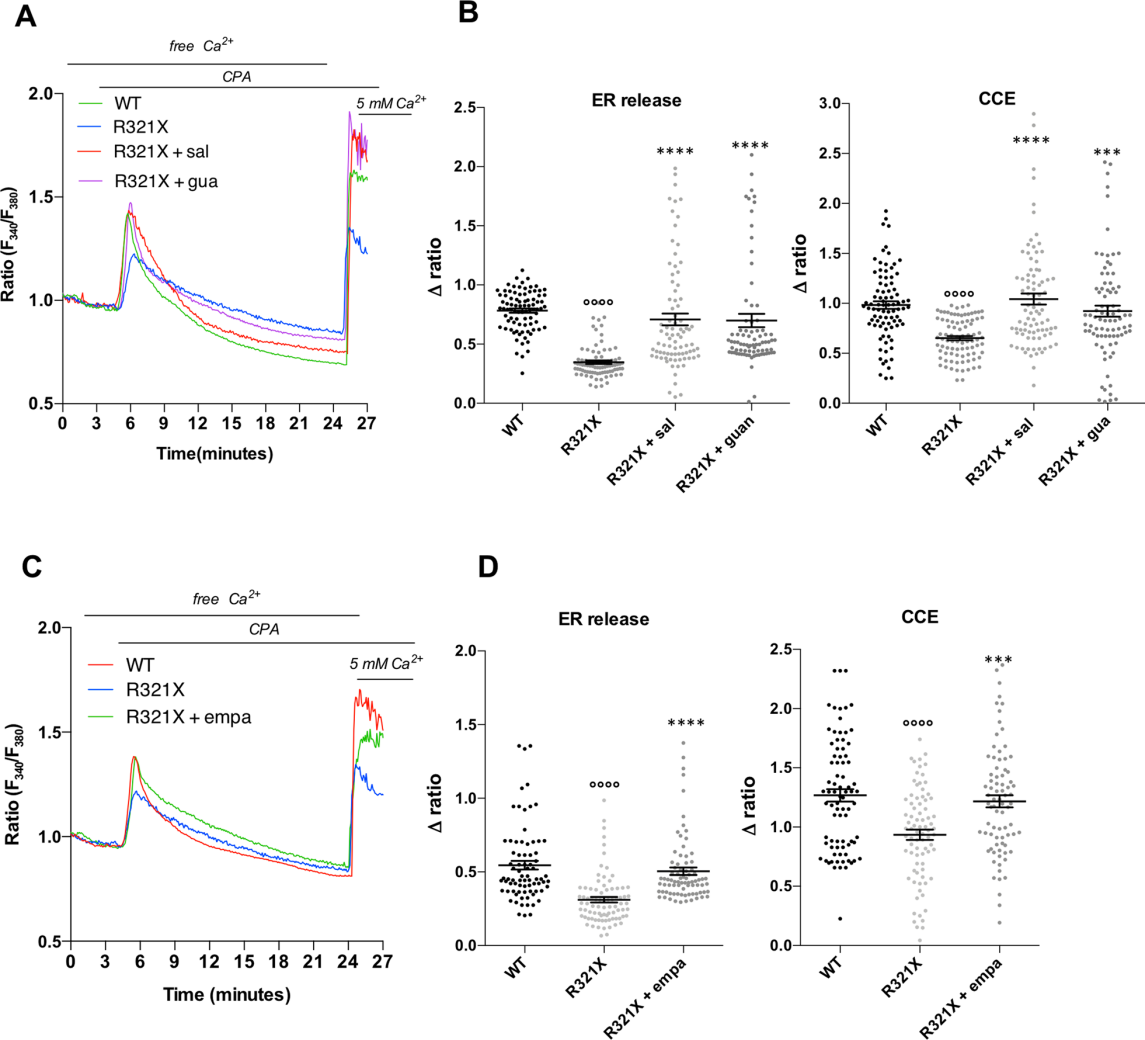
Effect of salubrinal, guanabenz and empagliflozin on cytosolic Ca^2+^ dynamics in LMNA R321X-cardiomyocytes.** A** Changes in fluorescence ratio in 4 representative Fura-2-loaded cardiomyocytes showing the effect of salubrinal and guanabenz. Each color line represents an individual cell and a specific cell line, as reported in the inset. Horizontal black lines report experimental conditions of the perfusion ringer (free Ca^2+^, CPA, 5 mM Ca^2+^). **B** Data plot summarizing the cytosolic Ca^2+^ response induced by either CPA (ER release) or by 5 mM Ca^2+^ (CCE) in the experimental conditions shown in A. Plots show mean value of ∆ ratio ± SEM of all responsive cells (*n* = 82/82 responsive cells, in 3 independent experiments). Ordinary one-way ANOVA and Tukey's multiple comparisons test was used for statistical analysis (°°°°p = 0.0001 for WT vs R321X; ****p = 0.0001 and ***p = 0.004 for untreated R321X vs treated R321X).** C** Changes in fluorescence ratio in 3 representative Fura-2-loaded cardiomyocytes showing the effect of empagliflozin. Each color line represents an individual cell and a specific cell line, as reported in the inset. Horizontal black lines report experimental conditions of the perfusion ringer (free Ca^2+^, CPA, 5 mM Ca^2+^). **D** Data plot summarizing the cytosolic Ca^2+^ response induced by either CPA (ER release) or by 5 mM Ca^2+^ (CCE) in the experimental conditions shown in ** C** (*n* = 80/80 responsive cells, in 3 independent experiments). Plots show mean value of ∆ ratio ± SEM of all responsive cells (*n* = 80/80 responsive cells, in 3 independent experiments). Ordinary one-way ANOVA and Tukey's multiple comparisons test was used for statistical analysis (°°°°p = 0.001 for WT vs R321X; ***p = 0.006, ****p = 0.0001 for untreated R321X vs treated R321X)

**Fig. 7:**
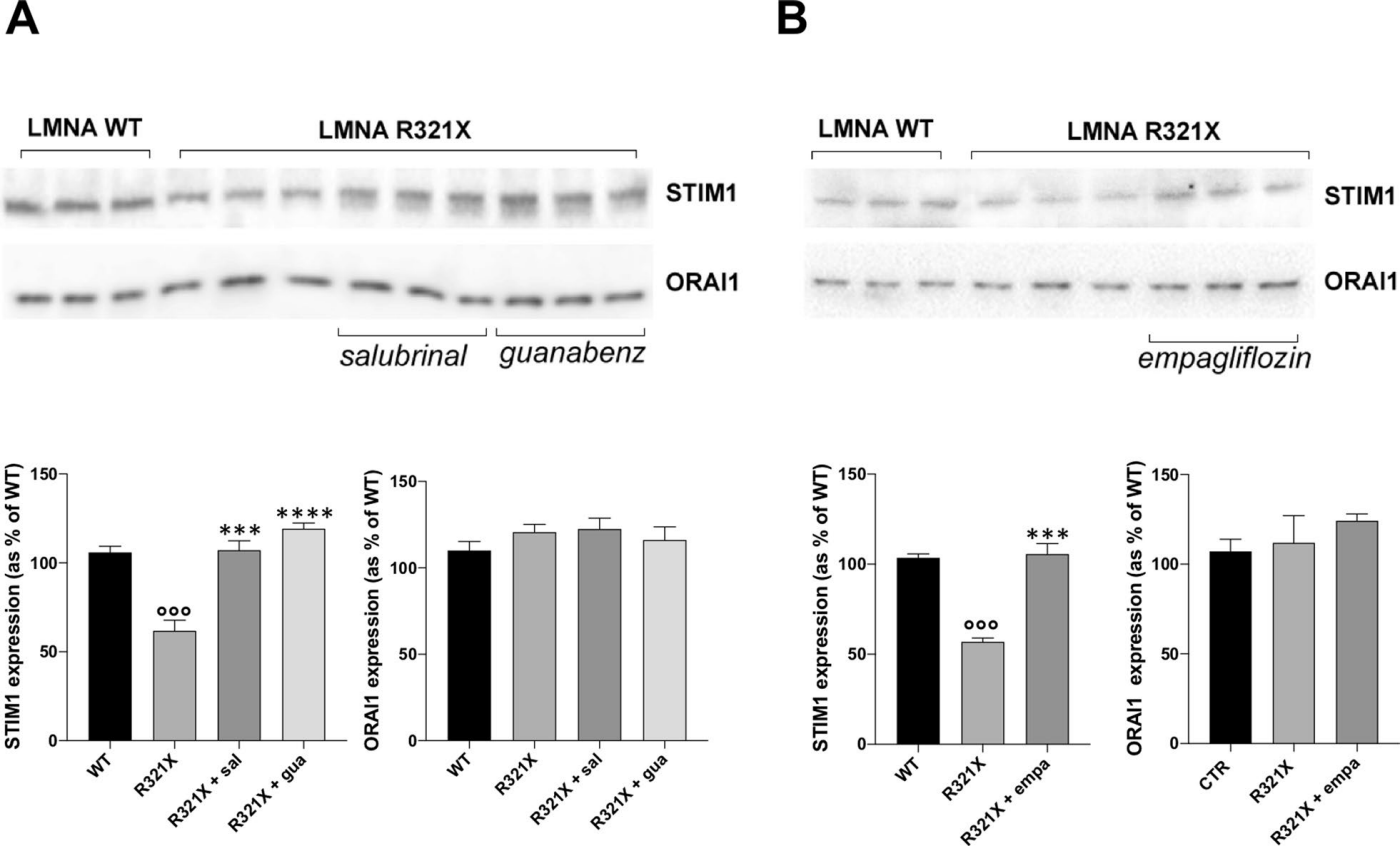
Effect of salubrinal, guanabenz and empagliflozin on STIM1 and ORA1 expression in LMNA R321X-cardiomyocytes. **A** Upper panel: Representative Western blotting of STIM1 ad ORA1 performed on lysates from LMNA R321X-cardiomyocytes treated with either 200 µM salubrinal or 20 µM guanabenz for 48 h and from LMNA WT-cardiomyocytes as control. The triplicate of each experimental conditions in the representative western blotting corresponds to three different biological replicas. Lower panel: densitometric analysis of the immunoreactive bands corresponding to STIM1 and ORA1, in the experimental conditions shown in the upper panel, normalized for total protein content and reported as % of the control condition (WT). Data are represented as mean ± SEM. Statistical analysis was performed on three independent experiments and significance calculated by one-way ANOVA and Tukey's multiple comparisons test (°°°p = 0.0001, for WT vs R321X; ***p = 0.0003, ****p = 0.0001 for untreated R321X vs treated R321X). **B** Upper panel: Representative Western blotting of STIM1 ad ORA1 performed on lysates from LMNA R321X-cardiomyocytes treated with either 10 µM empagliflozin for 48 h and from LMNA WT-cardiomyocytes as control. The triplicate of each experimental condition in the representative western blotting corresponds to three different biological replicas. Lower panel: densitometric analysis of the immunoreactive bands corresponding to STIM1 and ORA1, in the experimental conditions shown in the upper panel, normalized for total protein content and reported as % of the control condition (WT). Data are represented as mean ± SEM. Statistical analysis was performed on three independent experiments and significance calculated by one-way ANOVA and Tukey's multiple comparisons test (°°°p = 0.0001, for WT vs R321X; ***p = 0.0003, for untreated R321X vs treated R321X)

We finally analyzed the ability of ER to release and uptake Ca^2+^, which is a fundamental mechanism in the electromechanical coupling in cardiomyocytes. To this end we monitored cytosolic Ca^2+^ dynamics by Fura-2 upon ER Ca^2+^ depletion and the capacitative calcium entry (CCE) induction in the absence or presence of salubrinal, guanabenz and empagliflozin. When ER Ca^2+^ stores were depleted blocking the SERCA pump with 40 μM CPA in the absence of external Ca^2+^, cytosolic Ca^2+^ increased transiently because of the passive diffusion of Ca^2+^ through ER leaks (Fig. 6A, C, first pick). After complete ER Ca^2+^ emptying (about 20 min), re-addition of 5 mM Ca^2+^ in the extracellular solution in the continuous presence of CPA, rapidly increased cytosolic Ca^2+^ due to the CCE at the plasma membrane classically triggered by Ca^2+^ store depletion (Fig. 6A, C, second pick). The expression of LMNA R321X reduced both ER Ca^2+^ depletion and CCE when compared to their controls (Fig. 6A, C, blue lines). However, the amplitude of the two Ca^2+^ responses of the same protocol was recovered upon the treatment with either salubrinal or guanabenz (Fig. 6A, red line and purple line) as well as after empagliflozin treatment (Fig. 6C, green line). The quantitative analysis of the above-described Ca^2+^ dynamics are showed in Fig. 6B, D. These data clearly indicated an impaired release of Ca^2+^ from the ER, likely reflecting reduced ER Ca^2+^ content and increased [Ca^2+^]_cyt_ in LMNA R321X-cardiomyocytes, which was then rescued by all drugs tested. Reduced CCE in LMNA R321X-cardiomyocytes was also observed, which was also reversed by salubrinal, guanabenz and empligliflozin. However, the knowledge of mechanisms underlying this phenomenon has required more investigation. In vertebrate the CCE is triggered mainly by the ER Ca^2+^ sensor protein STIM1, which once activated by ER Ca^2+^ depletion, oligomerizes and accumulates at ER-plasma membrane junctions where it binds, traps and opens ORAI1 pore-forming Ca^2+^ channels thus refilling ER stores. We indeed checked for the expression levels of STIM1 and ORAI1 in LMNA WT and R321X-cardiomyocytes by Western blotting. As shown in Fig. 7, the expression of STIM1 was dawnregulated in LMNA R321X-cardiomyocytes compared to their controls whereas the expression levels of ORAI1 resulted comparable in the two lines of cardiomyocytes (Fig. 7A, B, LMNA WT and LMNA R321X). Interestingly, the expression levels of STIM1 were recovered upon all drugs treatment (Fig. 7A LMNA R321X + salubrinal and guanabenz; Fig. 7B, LMNA R321X + empagliflozin). The quantitative analysis of the above-described phenomena clearly showed both the significant decrease of STIM1 expression in LMNA R321X-cardiomyocytes and the efficacy of all drugs in restoring the expression levels of STIM1 in these cardiomyocytes (Fig. 7A, low panel, WT, R321X, R321X + sal, R321X + gua; Fig. 7B, low panel, WT, R321X, R321X + empa).

## Discussion

**Fig. 8:**
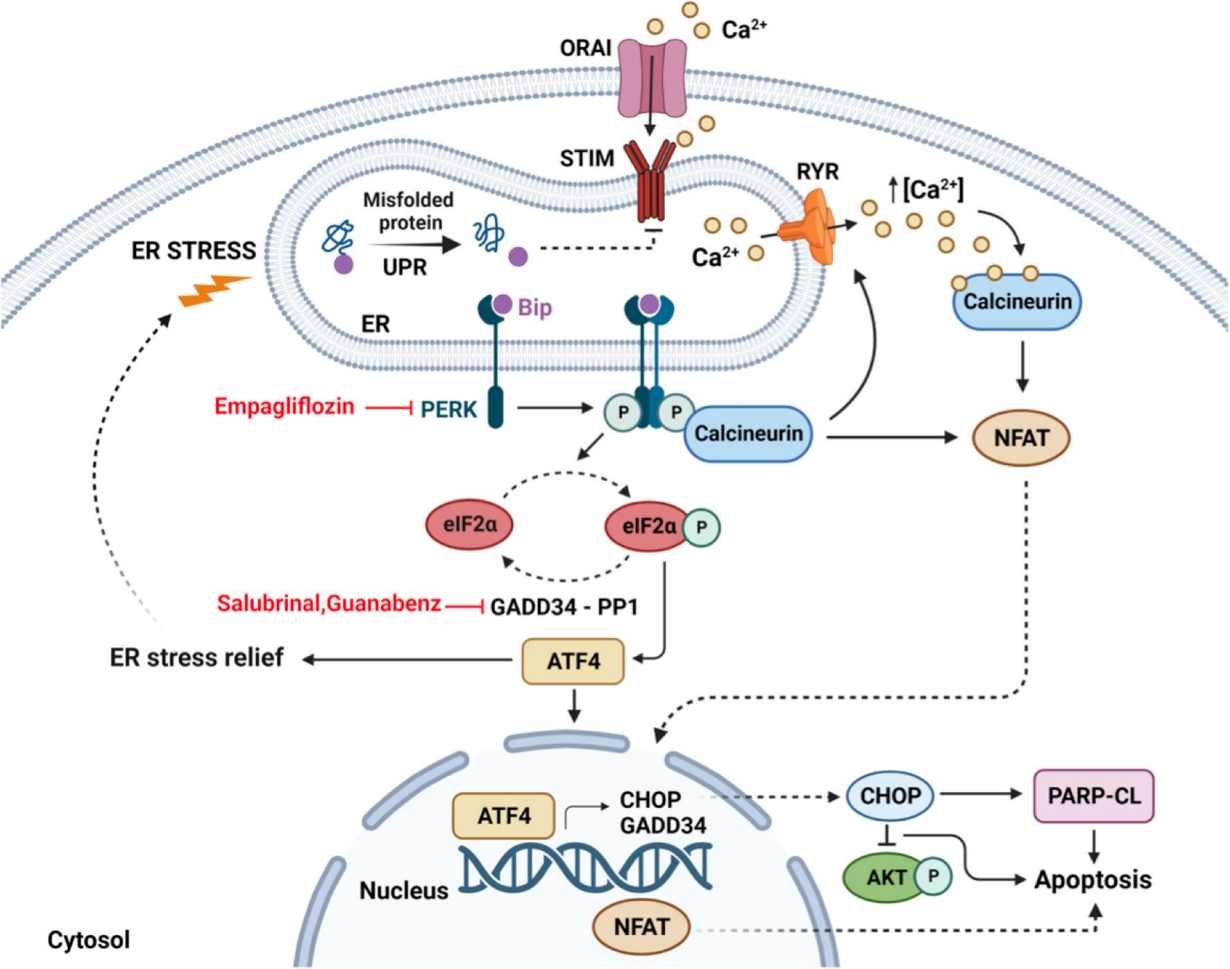
Proposed working model. The activation of PERK arm of UPR by PERK phosphorylation results in ER Ca^2+^ leak and increase in cytosolic Ca^2+^, most likely due to the activation of calcineurin and Ryanodine Receptors (RyR) from p-PERK. Concomitantly, STIM1 expression is downregulated into the ER membrane of LMNA R321X-cardiomyocytes, thus decreasing the Capacitive Ca^2+^ Entry in these cells, contributing the impairment in handling Ca^2+^(STIM-ORAI). The increase in cytosolic Ca^2+^ together with calcineurin activation induce NFAT translocation from cytosol to nuclei of these cardiomyocytes, most likely contributing to activation of pro-apoptotic pathways. In addition, PERK phosphorylation activates a downstream pathway: eiF2α phosphorylation (p-eiF2α) and ATF4 expression. ATF4 alleviates ER stress. If ER stress is persistent, ATF4 induces the expression of the pro-apoptotic factor CHOP and increases the apoptosis rate in LMNA R321X-cardiomyocytes, as shown by the caspase depended PARP-cleavage in these cardiomyocytes (PARP-CL). The activation of the CHOP-dependent pro-apoptotic pathway is also paralleled by the inhibition of the pro-survival pathway of AKT (p-AKT). Salubrinal and guanabenz act inhibiting eiF2α de-phosphorylation, sustaining ATF4 expression in the direction of ER stress alleviation. Empagliflozin, instead, acts directly inhibiting PERK activation. All drugs shutting down the PERK pathway of the UPR recover ER Ca^2+^ leaks, STIM1 downregulation, cytosolic Ca^2+^ overload and the downstream pro-apoptotic responses. The cartoon has been created with BioRender.com

This work represents the final step toward the definition of a possible personalized therapeutic approach in the field of cardiac laminopathies. We previously demonstrated that an Italian family affected by a severe DCM with history of sudden deaths at young age, carried a mutation in the *Lmna* gene encoding for a truncated variant of the LMNA, LMNA R321X. Interestingly, we demonstrated that such variant mis-localized and accumulated into the ER when expressed in mature cardiomyocytes, as expected from the absence of the nuclear localization sequence (NLS), inducing ER dysfunction and the up-regulation of pro-apoptotic markers. Of note, a very similar truncated variant of LMNA misplaced into the ER when expressed in cardiomyocytes but induced a severe cardiomyopathy by a different pathogenic mechanism [18]. These findings clearly indicate that each *Lmna* mutation drives cardiac detriment by its own and unique pathway and that ER stress induced by the accumulation of LMNA R321X into ER might be a useful pharmacological target in the LMNA R321X associated cardiac laminopathy. Specifically, we deciphered the pathways activated in LMNA R321X-cardiomyocytes, which we summarized in the cartoon of Fig. 8. Once activated the PERK arm of the UPR upon the accumulation of mis-folded LMNA R321X into the ER, this results in ER Ca^2+^ leak, most likely due to the activation of calcineurin and ryanodin receptors (RyR). Interestingly, PERK/calcineurin signaling has been identified in diabetic cardiomyopathy as novel pathway regulating the intracellular Ca^2+^ dynamics and involved in the pathogenesis of the disease [33]. Accordingly, we found an increased steady-state cytosolic Ca^2+^ level in LMNA R321X-cardiomyocytes, and the activation the calcineurin/NFAT pathway with NFAT translocation from cytosol to nuclei of these cardiomyocytes. Interestingly, calcineurin/NFAT pathway has been found activated in response to pathological stimulation in mice and humans, such as pressure overload or myocardial infarction and precedes the development of pathological cardiac hypertrophy, DCM and heart failure [34] thus suggesting a pivotal role in the heart function detriment when activated.

Concomitantly, STIM1 expression into ER was downregulated in LMNA R321X-cardiomyocytes, thus decreasing the Capacitive Ca^2+^ Entry (CCE), finally contributing to the ER impairment in maintaining luminal Ca^2+^ levels in these cells. The downregulation of STIM proteins with unaffected ORAI protein expression was already observed in cells under ER stress conditions [35, 36], suggesting an active role of this ER protein in the initiating/sustaining ER stress and UPR signaling.

The activation of PERK-dependent arm of the UPR, induces the phosphorylation of the downstream effector eIF2α, which reduced translation of misfolded proteins thus alleviating ER workload and promoting cell survival. As the stress declines, the GADD34 protein facilitates the dephosphorylation of eIF2α by recruiting the protein phosphatase PP1 restoring normal protein synthesis [37]. On the other hand, phosphorylation of eIF2α increases the expression of the transcription factor ATF4, which regulates a wide range of genes playing a crucial role in cell adaptation to stress conditions and cell survival. However, when ER stress persists p-eIF2α dephosphorylation increases again ER workload inducing a pro-apoptotic response, characterized by the expression of the pro-apoptotic factor CHOP and the caspase activation as demonstrated by the PARP cleavage. Interestingly, the activation of the CHOP-dependent pro-apoptotic pathway was also paralleled by the inhibition of the pro-survival pathway of AKT demonstrated by the decrease of AKT phosphorylation in LMNA R321X-cardiomyocytes compared to their controls. Of note, it has been demonstrated that one way by which CHOP may promote apoptosis under ER stress is inhibiting the phosphorylation and the activity of the pro-survival kinase AKT [38, 39], although we cannot exclude that other mechanisms are involved in the down-regulation of AKT pathway in LMNA R321X-cardiomyocytes.

Once dissected the pathways triggered in LMNA R321X-cardiomyocytes, we analyzed the effect of different drugs on these pathways. Interestingly, in 2005, Boyce and colleagues reported that a small molecule, salubrinal, acted as an inhibitor of the GADD34-PP1 complex, which dephosphorylates eIF2α [40]. Thus, salubrinal was supposed to weaken the synthesis of unfolded or misfolded proteins during persistent ER stress contributing to the preservation of homeostasis in ER and saving cells from apoptosis. Accordingly, we found that salubrinal, increasing the rate of eIF2α phosphorylation in LMNA R321X-cardiomyocytes and the expression of the downstream ATF4 factor, relieved the activation of PERK pathway, downregulated the expression levels of apoptosis markers such as CHOP and PARP-CL and restored the pro-survival ATK pathway. Salubrinal was previously found to be neuroprotective in animal models of Amiotrophic Lateral Sclerosis (ALS) [41], cerebral ischemia/reperfusion [42] and traumatic brain injury [43] where ER stress was involved in the pathogenesis of the disease. In addition, salubrinal was reported to protect cardiomyocytes against apoptosis in a rat model of myocardial infarction, where ER stress was triggered by hypoxia [44]. Despite the clear effect in counteracting ER stress in cell and animal models, salubrinal has never been further developed for the use in clinical practice, most likely because of its lack of selectivity for ER-stress related phosphatases and its unstable chemical structure, making its clinical use unfeasible [40].

In contrast, guanabenz, an FDA-approved α2-adrenergic receptor agonist formerly used in clinical practice for the treatment of hypertension [45], has been found to efficiently inhibit GADD34-PP1 complex independently from α2-adrenergic agonism [46]. Of note, guanabenz selectively inhibits only the stress-induced eIF2α phosphatase in stressed cells [46].

We found that guanabenz was efficient as salubrinal in restoring homeostasis in LMNA R321X-cardiomyocytes. Of note, HL-1 cardiomyocytes as well as human heart doesn’t express α2-adrenergic receptors [47, 48], thus confirming that guanabenz was acting on HL-1 cardiomyocytes independently from its α2-adrenergic agonism.

In agreements with our findings, guanabenz proved to efficiently rescue motoneurons function in in vitro and in vivo ALS models in which ER stress-related protein misfolding is central to the pathogenesis of the disease [49–51]. Based on these results in 2017 the ProMISe trial, Phase II clinial trial, has been started treating ASL patients with guanabenz [52].

Interestingly, guanabenz was found also to interfere with ER stress and to exert protective effects in cardiac myocytes and in 3-dimensional engineered heart tissue [53].

Owing to its role on the α2-adrenergic receptors and possible effects on the cardiovascular system as a result of its activation, guanabenz could seem unsuitable for patients affected by cardiomyopathies. In fact, α2-adrenergic receptors activation decreased centrally the sympathetic outflow and blood pressure. Interestingly, the first symptomatic treatment for DCM due to *Lmna* mutations, like that affecting the family expressing LMNA R321X, is based on the use of anti-hypertensive drug such as β-blockers and ACE inhibitors [4], thus suggesting that the use of guanabenz could be in line with the currently used therapeutic management of cardiac laminopathies.

Of note, several clinical trials with patients with type 2 diabetes mellitus (T2DM) treated with inhibitors of SLGT-2, generically called gliflozins, reported unexpected beneficial effects on cardiovascular outcomes in these patients, in the absence of significant adverse effects [54–57]. Subsequently, studies investigating the effect of gliflozins in patients with heart failure (HF) with either reduced or preserved ejection fraction and without T2DM were conducted. All of them demonstrated that gliflozins have a significant positive impact on HF and cardiac pathological remodeling independent from their anti-diabetic effects [58] or ejection fraction [59]. The current clinical guidelines for HF, therefore, now include SGLT2i as a Class 1A recommendation for the treatment of HF [60, 61]. Interestingly, gliflozins not only counteract cardiac remodeling but also arrhythmias. Although the mechanisms by which gliflozins act on the cardiovascular system have not been completely elucidated, their role in the protection against various cardiovascular diseases, especially HF, myocardial hypertrophy, myocardial infarction at the currently used dosage is undisputed.

Here we studied the ability of empagliflozin in counteracting ER stress in LMNA R321X- cardiomyocytes. Ten µM empagliflozin for 48 h was well tolerated by HL-1 cardiomyocytes and highly effective in reducing the expression of both CHOP and PARP-CL decreasing PERK phosphorylation levels and rescuing p-AKT levels. Our findings are in agreement with previous reports where gliflozins were able to inhibit the PERK-CHOP pathway of UPR acting on PERK activation itself, as shown in the brain of rat model of Parkinson's disease [28], in the heart of I/R injury mouse model [62] and in doxorubicin induced cardiac fibrosis and apoptosis in Streptozotocin (STZ) rats [63].

Interestingly, it has been demonstrated that in patients treated with dapagliflozin, compared with the untreated group, the risk of atrial fibrillation was significantly reduced a well as the total number of atrial fibrillation events [64]. Moreover, a meta-analysis published recently suggested that gliflozins specifically reduced the risk of ventricular tachycardia [65]. Of note, we previously shown that patients carrying the variant LMNA R321X are affected by DCM with recurrent atrial fibrillation and ventricular tachycardia [17]. Thus, the use of gliflozins in these patients may be useful not only for their ability to alleviate ER stress but also for their antiarrhythmic properties.

We would also like to underlie that all drugs tested on living LMNA R321X-cardiomycytes restore the ability of the ER to correctly store Ca^2+^ and to trigger CCE. Both the ER Ca^2+^ levels and CCE in cardiomyocytes are essential in cardiomyocytes electrical and mechanical events [66, 67].

## Conclusion

Unlike most other forms of familial cardiomyopathies, sudden cardiac death may be the first manifestation of LMNA cardiomyopathy, even in the absence of systolic dysfunction, because of malignant arrhythmias such as ventricular tachycardia and fibrillation. Current therapy for LMNA cardiomyopathy is only symptomatic and follows the standard heart failure regimen thus making the treatment of LMNA cardiomyopathy a challenging field in the cardiovascular research and clinic. Our findings clearly demonstrated that ER stress due to LMNA R321X accumulation into the ER of cardiomyocytes was involved in the pathogenic mechanisms of the associated severe DCM. Interestingly, we found that ER stress in LMNA R321X-cardiomyocytes can be counteracted basically in two ways: (a) sustaining an adaptive UPR using molecules such as salubrinal and guanbenz or (b) blocking a maladaptive UPR with gliflozins such as empagliflozin. Some of these drugs, guanabenz and empagliflozin, are already used in the clinical practice, thus providing preclinical evidence for a possible ready-to-use therapeutic strategies in the patients expressing LMNA R321X pathogenic variant.

## Data Availability

The datasets used and/or analyzed during the current study are available from the corresponding author on reasonable request.

## Abbreviations

LMMA: Lamina A/C protein
DCM: Dilated cardiomyopathy
ER: Endoplasmic reticulum
UPR: Unfolded protein response
IRE1: Inositol-requiring protein 1
PERK: Protein kinase RNA-like ER kinase
ATF6: Activating transcription factor 6
eIF2α: Eukaryotic translation initiation factor 2 alpha
ATF4: Activating transcription factor 4
CHOP: CCAAT-enhancer-binding protein homologous protein
s-Xbp1: Spliced X-box-binding protein-1
STIM: Stromal interaction molecule
ORAI: Calcium release-activated calcium channel protein
GRP78: Glucose-regulated protein 78
GADD34: DNA damage-inducible 34
PP1: Protein phosphatase 1
PARP-CL: Cleaved poly(ADP-ribose) polymerase 1
CCE: Capacitative calcium entry
ALS: Amiotrophic lateral sclerosis
SGLT-2: Sodium-glucose co-transporter-2
T2DM: Type 2 diabetes mellitus
HF: Heart failure
